# Oligosaccharide Ligands of Galectin-4 and Its Subunits: Multivalency Scores Highly

**DOI:** 10.3390/molecules28104039

**Published:** 2023-05-11

**Authors:** Kristýna Slámová, Jakub Červený, Zuzana Mészáros, Tereza Friede, David Vrbata, Vladimír Křen, Pavla Bojarová

**Affiliations:** 1 Laboratory of Biotransformation, Institute of Microbiology of the Czech Academy of Sciences, Vídeňská 1083, 142 00 Prague 4, Czech Republic; slamova@biomed.cas.cz (K.S.); cervenjaku@seznam.cz (J.Č.); kren@biomed.cas.cz (V.K.); david.vrbata@biomed.cas.cz (D.V.); zuzana.meszaros@biomed.cas.cz (Z.M.); tereza.friedova@gmail.com (T.F.); 2 Department of Analytical Chemistry, Faculty of Science, Charles University, Hlavova 8, 128 43 Prague 2, Czech Republic; 3 Department of Biochemistry, University of Chemistry and Technology Prague, Technická 6, 160 00 Prague 6, Czech Republic; 4 Department of Biochemistry, Faculty of Science, Charles University, Hlavova 8, 128 43 Prague 2, Czech Republic

**Keywords:** blood-group antigen, inhibitor, galectin-4, multivalency, oligosaccharide, transglycosylation

## Abstract

Galectins are carbohydrate-binding lectins that modulate the proliferation, apoptosis, adhesion, or migration of cells by cross-linking glycans on cell membranes or extracellular matrix components. Galectin-4 (Gal-4) is a tandem-repeat-type galectin expressed mainly in the epithelial cells of the gastrointestinal tract. It consists of an N- and a C-terminal carbohydrate-binding domain (CRD), each with distinct binding affinities, interconnected with a peptide linker. Compared to other more abundant galectins, the knowledge of the pathophysiology of Gal-4 is sparse. Its altered expression in tumor tissue is associated with, for example, colon, colorectal, and liver cancers, and it increases in tumor progression, and metastasis. There is also very limited information on the preferences of Gal-4 for its carbohydrate ligands, particularly with respect to Gal-4 subunits. Similarly, there is virtually no information on the interaction of Gal-4 with multivalent ligands. This work shows the expression and purification of Gal-4 and its subunits and presents a structure–affinity relationship study with a library of oligosaccharide ligands. Furthermore, the influence of multivalency is demonstrated in the interaction with a model lactosyl-decorated synthetic glycoconjugate. The present data may be used in biomedical research for the design of efficient ligands of Gal-4 with diagnostic or therapeutic potential.

## 1. Introduction

Galectins (Gal-) form a small group of β-galactoside-binding lectins comprising fifteen members in humans. Their functions include the modulation of vital cellular processes, such as cell proliferation, apoptosis, adhesion, or migration by cross-linking glycans on cell membranes and/or extracellular matrix components [1]. Compared to Gal-1 and Gal-3, which have been widely shown to play significant roles especially in cancer progression, inflammation, fibrosis, heart disease, and stroke, as well as some metabolic disorders [2,3], there are few studies on the pathophysiology and the binding preferences of galectin-4 (Gal-4) [4]. Gal-4 is a tandem-repeat galectin consisting of two different carbohydrate-recognition domains (CRDs) at the N- and C-terminus (Gal-4N, and Gal-4C, respectively), each with distinct binding specificities, which are covalently linked with a specific peptide [5]. The crystal structures of native Gal-4 and its individual CRDs show that both CRDs contain a concave binding site with five subsites (A–E) and preferentially bind lactose in the D/E subsites. These extended subsites can further accommodate various glycan epitopes, depending on subtle interactions with specific amino acids [6,7,8].

Human Gal-4 is mainly expressed in the gastrointestinal tract, where it stabilizes lipid rafts [9] and delivers or recruits glycoproteins to lipid rafts [10] at the apical brush-border membranes of enterocytes. Furthermore, Gal-4 can crosslink sulfated glycosphingolipids and *N*- or *O*-glycosylated proteins with blood-group antigens by its CRDs to stabilize these rafts [11,12]. The role of Gal-4 in malignant tumor progression and metastasis is variable and depends on the cancer type. Its reduced expression in tumor tissue was found in patients with colon cancer [13,14]. On the other hand, its expression was observed to be significantly increased in the sera of colorectal cancer patients, especially in those with liver metastases, compared with a healthy control group [15,16]. The discrepancy between the levels of Gal-4 in the tumor tissues and in the blood sera of patients can be explained by the extensive production of circulating Gal-4 by many other types of cell, such as peritumoral stromal cells and a variety of immune cells [15]. Moreover, Gal-4 expression is correlated with increased metastasis and cancer progression in liver and lung cancers [17,18].

Furthermore, Gal-4 was reported to bind to glycosphingolipid-based glycans of the lacto-, type 1 (lacto-*N*-tetraose, LNT), and type 2 (lacto-*N*-neotetraose, LN*n*T) series, as well as to the core 1-*O*-glycan motif. In addition, Gal-4 showed binding to blood-group ABH antigens and sulfated glycans bearing 3-*O*-sulfated galactosyl [19,20]. Regarding native glycoproteins, Gal-4 was shown to bind to asialofetuin and mucins. Sialylated glycoproteins were not bound, although 3′-*O*-sialylated LacNAc type 1 and type 2 were recognized [20]. Both CRDs of Gal-4 exhibit distinct glycan-binding preferences. Studies using X-ray crystallography and molecular dynamics simulation indicated that both CRDs prefer blood-group antigen A; in contrast, Gal-4N displayed a significantly higher binding affinity to sulfated glycans than Gal-4C. The binding constants of blood-group antigen A with Gal-4N and Gal-4C determined by fluorescence anisotropy assay were 164 and 75 µM, respectively [6,7]. Recently, the binding preferences of Gal-4N for various types of blood-group antigens A and B were studied in detail by employing NMR, ITC, and molecular modeling. The results demonstrated that the highest binding affinity of Gal-4N was to galactosyl-capped tetrasaccharide antigen B, closely followed by GalNAc-capped antigen A [21]. A recent study revealed the binding of Gal-4N and Gal-4C to desialylated complex *N*-glycans on glycan-engineered CHO cells [22]. In general, very few publications provide more accurate information than a relative comparison of various carbohydrate ligands by solely reporting IC_50_ or *K*_D_ values. Similarly, there is virtually no information on the effects of the multivalent presentation of glycan epitopes, except for a single study on natural *O*-glycosylated mucin-type glycoproteins presenting ABH blood groups [23]. To the best of our knowledge, no other studies with glycan ligands have been published that demonstrate the positive effect of multivalent presentation on Gal-4 binding.

Moreover, in the few existing studies on the affinity of carbohydrate ligands to Gal-4 and its CRDs, the authors use different methods, and there are no studies available that compare the assays of native Gal-4 and its subunits using the same methods and ligands. In this work, we present the expression of a His-tagged construct of human Gal-4 and its two subunits, Gal-4N and Gal-4C, and a structure–affinity relationship study with a library of selected oligosaccharide ligands performed by using the competitive ELISA method. Furthermore, the impact of multivalency on the interaction of whole Gal-4 and its subunits is presented with a model lactosyl-decorated neo-glycoprotein. The obtained data significantly expand the available information on potential ligand candidates, which are useful in biomedical research on Gal-4.

## 2. Results

### 2.1. Expression and Purification of Gal-4 and Its Binding Domains

**Figure 1:**
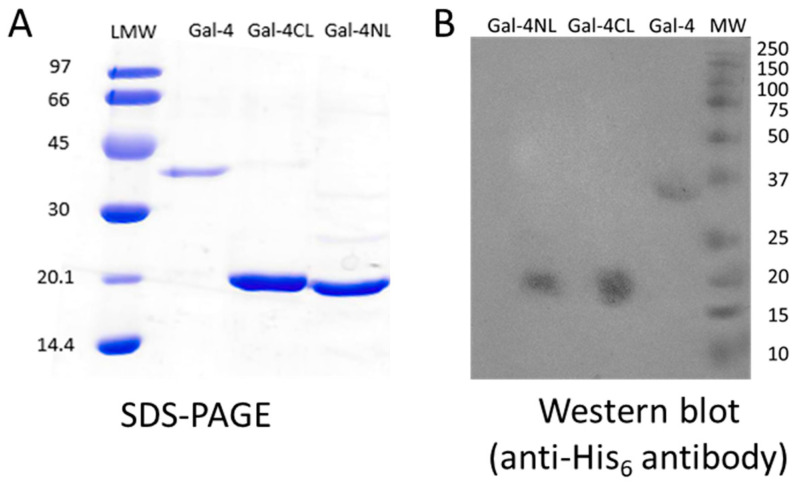
SDS-PAGE (**A**) and Western blot (**B**) analysis of isolated (from left to right) Gal-4NL (17.9 kDa), Gal-4CL (17.1 kDa), and Gal-4 (35.9 kDa). LMW—Amersham Low-Molecular-Weight Calibration Kit for SDS Electrophoresis (GE Healthcare, Chicago, IL, USA); Anti-His_6_ antibody (His-probe, Santa Cruz Biotechnology, Dallas, TX, USA); MW standard—Precision Plus Protein™ Kaleidoscope™ Prestained Protein Standards (Bio-Rad, Hercules, CA, USA).

The Gal-4 construct carrying an N-terminal His_6_-tag cloned into the pET28a vector was expressed in *Escherichia coli* Rosetta (DE3) pLysS cells and purified to homogeneity by metal-ion-affinity chromatography (Figure 1). Furthermore, the N-terminal and C-terminal subunits of Gal-4, designated as Gal-4NL (aa 1-160), and Gal-4CL (aa 169-323), respectively, were expressed and purified using analogous procedures. Both subunits carried a 10-amino-acid linker at their respective ends, interconnecting these domains in the native protein. The presence of the linker was necessary to preserve the lectin activity of the subunits. Notably, while the whole Gal-4 remained stable and active at 4 °C for ca 3 weeks, the stability of its subunits was significantly lower, ca one week. After this time, there was a significant loss of binding activity. The typical yields of the purified forms of Gal-4 are listed in Table 1. The production yields of individual Gal-4 subunits, expressed as mg of protein gained from one gram of *E. coli* cells, were about one half of those obtained for the whole Gal-4 construct, which suggests that the molar yields of these proteins were very similar. The purity of the proteins was confirmed by SDS-PAGE (Figure 1A); the presence of the His-tag was confirmed by Western blot, using detection with anti-His_6_-tag antibody (Figure 1B).

### 2.2. Carbohydrate Ligands of Gal-4

**Figure 2:**
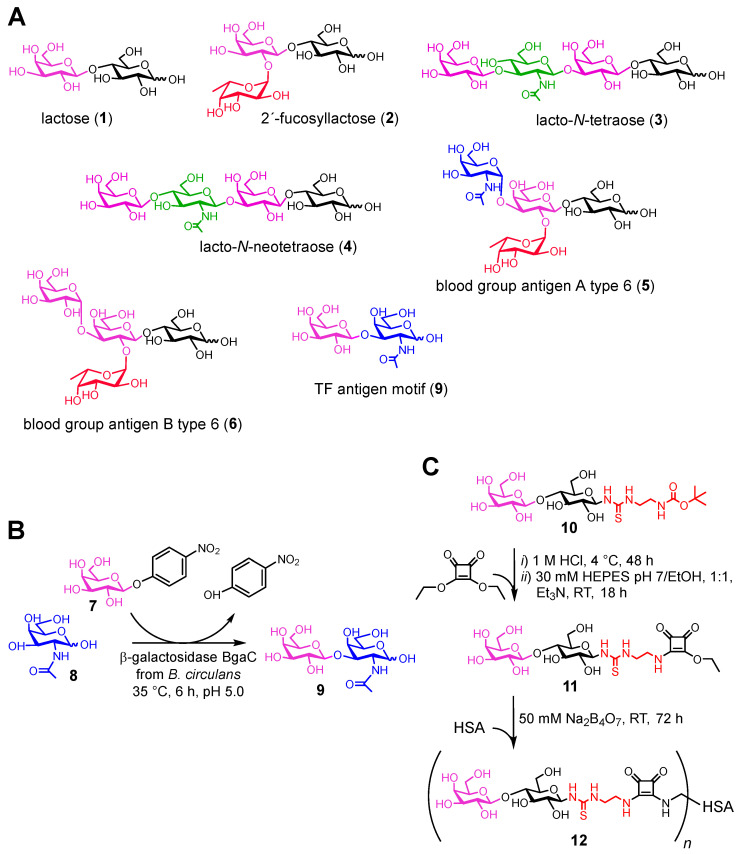
(**A**) Carbohydrate ligands of Gal-4 used in this study: lactose (**1**; Galβ4Glc), 2′-fucosyllactose (**2**, Fucα2Galβ4Glc), lacto-*N*-tetraose (**3**, Galβ3GlcNAcβ3Galβ4Glc); lacto-*N*-neotetraose (**4**, Galβ4GlcNAcβ3Galβ4Glc); blood-group antigen A type 6 (**5**, GalNAcα3[Fucα2]Galβ4Glc); blood-group antigen B type 6 (**6**, Galα3[Fucα2]Galβ4Glc); TF antigen motif (**9**, Galβ3GalNAc). (**B**,**C**) The synthetic routes towards (**B**) TF antigen, and to (**C**) lactosyl-decorated neo-glycoprotein. HSA, human serum albumin.

The library of carbohydrate ligands used in this study (**1**–**6**, **9**; Figure 2A) comprised commercially available oligosaccharides that have either been previously reported to have affinity to Gal-4 or are typical ligands of other, more thoroughly studied galectins, such as Gal-1 or Gal-3 [3,6,7,20,21]. Ligand **9**, the Thomsen–Friedenreich antigen structural motif (TF antigen; Galβ3GalNAc [24]), was prepared in a good yield by enzymatic synthesis using recombinant wild-type β-galactosidase BgaC from *Bacillus circulans* [25]. This enzyme, particularly in its mutant glycosynthase form, was previously used for the selective β(1→3)-galactosylation of various acceptors [26,27]. However, the selective preparation of the free TF antigen motif by the wild-type enzyme has not been reported, to our knowledge. By using the commercially available priceworthy *p*NP-Gal donor and galactose acceptor, this ligand was prepared in one step, at a yield of 51%, and structurally characterized (Figure 2B).

In addition, a synthetic lactosyl-decorated neo-glycoprotein was included in the studied carbohydrate-ligand library. This multivalent compound was prepared according to the previously described method, by conjugating the lactosyl moiety, functionalized at C-1 with an amino group, to a human-serum-albumin carrier via diethyl-squarate linkage (Figure 2C). The thiourea linker adjacent to the carbohydrate was previously proven to be a practical tool for multivalent presentations, with a variety of scaffolds [28,29], and to be highly compatible with enzymatic glycosylations [30]. The *t*Boc-protected amino-functionalized lactose ligand **10** (lactosyl-*t*Boc) was prepared through a process analogous to the established procedure for *t*Boc-protected amino-functionalized GlcNAc (GlcNAc-*t*Boc) at an overall yield of 23%, and structurally characterized [31]. The resulting neo-glycoprotein (MW 72.2 kDa) was analyzed by MALDI-TOF mass spectrometry, and the number of attached lactosyl residues was established as 9.6. Therefore, we were also able to determine the relative potency (*rp*) and relative potency per lactosyl (*rp*/*lactosyl*) of the binding to Gal-4, as indicated in Table 2 below.

### 2.3. Binding Affinity of Gal-4 and Its Subunits to a Library of Carbohydrate Ligands

The affinities of purified His-tagged Gal-4 and its binding subunits Gal-4NL and Gal-4CL with a library of seven oligosaccharides (**1**-**6**; **9**) and one defined multivalent lactosyl-decorated neo-glycoprotein (**12**) were investigated using a competitive enzyme-linked immunosorbent assay (ELISA).

**Figure 3:**
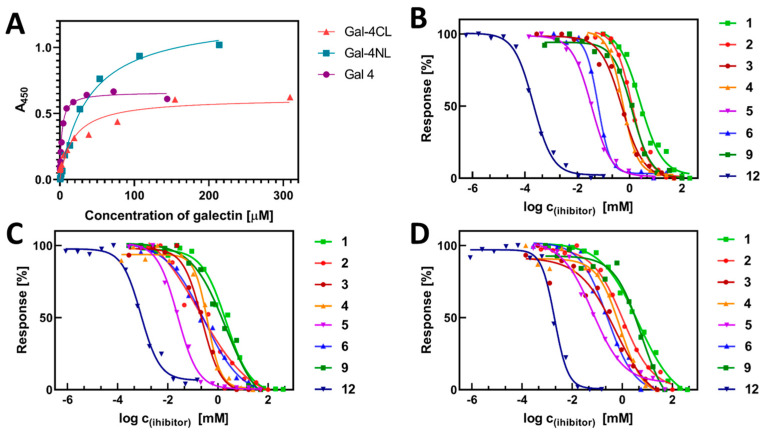
(**A**) The curves of dose–response direct binding of whole Gal-4 protein and its subunits, Gal-4NL and Gal-4CL, to immobilized standard ligand asialofetuin (ASF). *K*_D_ (whole Gal-4) = 3.3 ± 1.1 µM; *K*_D_ (Gal-4NL) = 38 ± 3 µM; *K*_D_ (Gal-4CL) = 19 ± 2 µM. (**B**) The curves of dose–response inhibition of binding of the whole Gal-4 to immobilized ASF by monovalent inhibitors **1**–**6** and **9**, and by multivalent neo-glycoprotein inhibitor **12**. Respective values are in Table 2. (**C**) The curves of dose–response inhibition of binding of the Gal-4NL subunit to immobilized ASF by monovalent inhibitors **1**–**6** and **9**, as well as by multivalent neo-glycoprotein inhibitor **12**. Respective values are in Table 2. (**D**) The curves of dose–response inhibition of binding of the Gal-4CL subunit to immobilized ASF by monovalent inhibitors **1**–**6** and **9**, as well as by multivalent neo-glycoprotein inhibitor **12**. Respective values are in Table 3.

To design the ELISA method, we first examined the direct binding of the whole Gal-4, Gal-4NL, and Gal-4-CL to standard glycoprotein asialofetuin (ASF, MW 48.4 kDa), which carries up to nine LacNAc epitopes. The ASF was immobilized by hydrophobic interaction in microtiter-plate wells, and the binding of increasing concentrations of Gal-4 proteins was quantified by colorimetric immunodetection using horseradish peroxidase conjugated to an anti-His-tag antibody. The apparent dissociation constants (*K*_D_) were calculated from the non-linear regression of the binding curves (Figure 3). The total Gal-4 protein reached a low micromolar range of apparent *K*_D_ with ASF (3.3 ± 1.1 µM), which was comparable to the values of Gal-1 and Gal-3 determined by the same method [32]. In contrast, individual Gal-4 subunits showed *K*_D_ values that were about one order of magnitude higher (*K*_D_ = 38 ± 3 µM and 19 ± 2 µM for Gal-4NL and Gal-4CL, respectively). These notable differences between the affinities of the subunits with ASF also resulted in slightly different setups for the competitive ELISA assays. While for the Gal-4 ELISA measurement we used the concentration of Gal-4 of 2.5 μM, for both subunits, it was 25 μM. Thus, we produced saturated inhibitory curves and acquired comparable values for the standard inhibitor, lactose, with all three protein constructs: IC_50_ = 2.5 ± 0.2 mM for the whole Gal-4 (Table 2); 2.9 ± 0.4 mM for Gal-4NL; and 8.6 ± 1.1 mM for Gal-4CL (Table 3). The potential of glycans **1**-**6**, **9** and neo-glycoprotein **12** to inhibit the binding of the Gal-4 to the immobilized ASF was essentially analyzed as described previously [32,33]. The Gal-4 proteins were incubated with increasing concentrations of the respective inhibitors, and the concentration-dependent inhibition of the binding to the immobilized ASF was quantified using spectrophotometric colorimetric immunodetection (HRP-conjugated anti-His-tag antibody). The respective half-maximal inhibition constants (IC_50_) were calculated from the non-linear regression of the sigmoidal curves (Figure 3), and they are shown in Table 2 and Table 3.

As shown in Table 2, the inhibitory potential of the tested monovalent glycans toward the whole Gal-4, expressed as IC_50_, ranged over more than two orders of magnitude (2.5 mM to 50 μM), depending on the glycan structure. The best inhibitors, blood-group antigens A and B, offered improvements in the inhibitory potency of up to 48-fold compared with the lactose standard. For both subunits, the structure-affinity relationship was even more distinct—the best inhibitor, blood-group antigen A, reached 130-fold and 320-fold increases in inhibitory potency with Gal-4NL and Gal-4CL, respectively, compared with the lactose. Blood-group antigen B was a considerably weaker inhibitor of the Gal-4 subunits, with 11-fold and 28-fold improvements over lactose for Gal-4NL and Gal-4CL, respectively. The IC_50_ values for the lacto-*N*-tetraose (**3**) were in the high micromolar range for both the whole Gal-4 and its subunits, Gal-4NL and Gal-4CL (*cf*. 0.43, 0.21 and 0.47 mM, respectively), closely followed by lacto-*N*-neotetraose (4) (*cf*. 0.6, 0.4, and 0.8 mM, respectively). Notably, the inhibitory potency of 2′-fucosyllactose (**2**) was much more distinct in the cases of both subunits (with up to 7.8-fold improvements in inhibitory potency compared with the lactose) than in the case of the whole Gal-4 (which was comparable to lactose). With all three proteins, the TF antigen motif (**9**) was a quite weak inhibitor, comparable to lactose.

**Figure 4:**
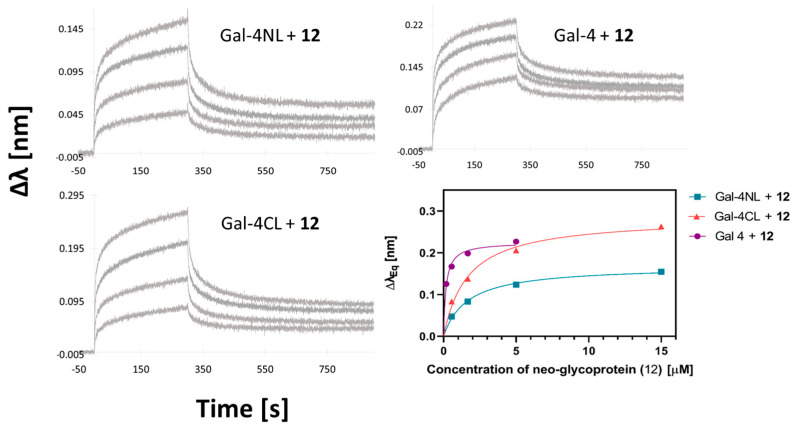
BLI sensograms of interaction between neo-glycoprotein **12** and Gal-4NL (*K*_D_ = 1.0 ± 0.2 µM), Gal-4CL (*K*_D_ = 1.7 ± 0.1 µM), and the whole Gal-4 (*K*_D_ = 0.17 ± 0.02 µM). The bottom-right panel shows the results of the steady-state analysis of the binding curves.

This work demonstrates, for the first time, an exciting increase in inhibitory potency achieved with a synthetic multivalent ligand by the multivalent presentation of the relatively weak inhibitor, lactose. The multivalent neo-glycoprotein **12** broke the micromolar border with the whole Gal-4, reaching an IC_50_ of 190 nM (a 1400-fold-stronger affinity with one bound lactosyl than the free lactose). For both subunits, the multivalency effect was also considerable (IC_50_ = 900 nM for Gal-4NL and 1.7 μM for Gal-4CL). This clearly demonstrates the strong beneficial effect of the multivalent presentation on Gal-4, as a representative of tandem-repeat galectins. To corroborate these results, an alternative affinity-determination method, biolayer interferometry (BLI), was used to assess the affinity of the whole Gal-4 and its both subunits with neo-glycoprotein **12** (Figure 4). In contrast to competitive ELISA, BLI is a direct affinity measurement that was already proven to be very useful for assessing the kinetics of interactions between galectins and their multivalent ligands [32]. However, this is the first time it was employed with Gal-4. The His-tagged fusion proteins were attached to the Ni-NTA biosensors by nickel chelation. The acquired kinetic data were subjected to steady-state analysis, and the shift of the interference pattern was plotted against the ligand concentration. In accordance with the ELISA results, neo-glycoprotein **12** exhibited the highest affinity for the whole Gal-4 (*K*_D_ = 0.17 ± 0.02 µM), followed by Gal-4NL (*K*_D_ = 1.0 ± 0.2 µM) and Gal-4CL (*K*_D_ = 1.7 ± 0.1 µM). These submicromolar values of *K*_D_ confirm the high potential of multivalency systems, such as neo-glycoprotein **12**, to inhibit Gal-4.

The affinity results (Table 2 and Table 3) showed relatively minor differences between the whole Gal-4 and its subunits. Therefore, we decided to obtain more insights into the potential aggregation behavior of the Gal-4 subunits in solution, which, theoretically, might have contributed to the multivalency effect, even with the monovalent Gal-4 subunits. The hydrodynamic radii of the whole Gal-4 and its subunits in the concentrations used in the ELISA assay (25 µM) were assessed using dynamic light scattering (DLS). This method provided information about the size distribution of the particles in the samples (Table 4). Notably, the samples of the Gal-4 were quite polydisperse, and the autocorrelation function fits commonly yield multimodal intensity-weighed size distributions. Therefore, the larger particles seemed to be more abundant in these distribution diagrams because light scattering is proportional to the sixth power of the scattering-matter diameter. To avoid misinterpretation, we also present the volume-weighed size distributions in Table 4, which highlight the most abundant populations of particles. These values indicate that in the cases of the whole Gal-4 and Gal-4CL, the large aggregates at 1200 nm and 400 nm, respectively, can be neglected (the volume-weighted size distributions accounted for 9 and 7 nm, respectively). The whole Gal-4 formed the most abundant particles, of 11 nm, which was similar to Gal-4CL with 8 nm particles. In the case of Gal-4NL, the solution was more polydisperse, and two significant populations were observed. The first population, of 1.3 nm, probably represented the individual Gal-4NL subunits, whereas the slightly larger population of 16 nm belonged to the aggregated species. These data suggest that in 25 µM concentrations, the Gal-4CL subunit tended to aggregate, whereas the Gal-4NL subunits remained in equilibrium with a major population of monomers and a minor population of aggregates, which were similar to those found with the other two proteins.

## 3. Discussion

The present work provides a unique insight into the binding affinities/inhibitory potencies of a representative series of oligosaccharides (seven compounds) using an easily implementable and reproducible competitive ELISA method. Since our results with individual subunits of Gal-4 revealed a considerable difference in binding to the competitor ligand ASF (with more than 10-fold-higher apparent *K*_D_s compared with the total Gal-4), we reflected this fact in the optimized setup of the competitive ELISA assay. The structure-affinity relationship study allowed the evaluation of the selectivity and behavior of the scarcely studied but biomedically very relevant tandem-repeat galectin—Gal-4. Previous studies generally worked on the principle of relative affinities, which hardly allows any comparison with the results obtained by other research groups [20]. However, the trends found herein and the relative order of the inhibitors according to their potency (in particular, lactose > 2′-fucosyllactose > lacto-*N*-tetraose > lacto-*N*-neotetraose > blood group antigens) were quite well in line with the affinity information available in the literature [6,7]. In agreement with our data, the relative order of affinities shown by Vokhmyanina et al. also identified lacto-*N*-tetraose (**3**) as a superior ligand to lacto-*N*-neotetraose (**4**), using flow cytometry analysis [20]. To our knowledge, the most thorough study giving a numerical assessment of binding affinities is the paper by Quintana et al., presenting *K*_D_s determined by isothermal titration calorimetry (ITC) and by NMR [21]. The *K*_D_ values reported therein for blood-group antigens A type 6 (**5**) and B type 6 (**6**) and Gal-4NL (other forms of Gal-4 were not analyzed) were in the micromolar range, as were our IC_50_ values, which were acquired by ELISA (*cf*. Table 2 with *K*_D_ = 86 μM for antigen A, and 51 μM for antigen B by ITC). However, in contrast to Quintana et al. [21], our results clearly identified blood-group antigen A as a superior inhibitor of Gal-4NL (a 12-fold stronger inhibitor than B). In our study, both the individual Gal-4NL and Gal-4CL subunits showed a significantly stronger affinity to the blood-group antigen A than to B (Table 3). The preference for A over B was also apparent, albeit less distinctively, in the affinities of the whole Gal-4 (Table 2). This discrepancy between our results and those of Quintana et al. [21] may have been caused by the use of completely different affinity-determination methods (in-solution vs. solid-phase assay). In sum, although all these comparisons are rather rough and relative (because the assessment methods used were different), they identify ELISA as a reliable method for assessing Gal-4 affinity.

Although size-related data for tandem-repeat galectins or their subunits (ca. 36 kDa and ca. 17 kDa, respectively) are currently lacking in the literature, the tendency of galectins to aggregate into dimers or oligomers was previously reported for other galectins, especially Gal-1 and Gal-3 [34,35]. Therefore, the generally relatively minor differences between the affinities found for the whole (bivalent) Gal-4 and its (monovalent) subunits might have partially contributed to the ability of the subunits to aggregate into larger clusters. However, the DLS analysis showed that this aggregation was relevant only for the Gal-4CL subunit, as the Gal-4NL subunit had an abundant population of monomers. Thus, the increase in the affinity of the Gal-4 subunits with the multivalent ligands was not primarily due to aggregation. This conclusion is neatly correlated with the results of the BLI measurements. Although aggregation cannot occur there because galectins are individually immobilized on the biosensor, the *K*_D_ results acquired by BLI for neo-glycoprotein **12** compared well with the ELISA data. The phenomenon of aggregation, reported here for the first time with Gal-4, deserves more thorough exploration in the future.

In addition, the present work addresses a hitherto rather neglected aspect of the affinity of Gal-4 to a multivalent synthetic ligand. Due to the structure of Gal-4, a tandem-repeat galectin with two binding sites, the multivalency effect was extreme, with improvement in binding of more than three orders of magnitude (1400-fold) when one lactosyl bound to the multivalent neo-glycoprotein was compared with free lactose. There was no positive contribution of the thiourea linker in this case, as the deprotected lactosyl amine showed IC_50_ = 2.8 mM (see also the legend in Table 2), which was practically the same value as that of the free lactose (2.5 mM). This was also in accordance with our previous results with other galectins and a variety of linkers and carriers [32,36,37]. Hence, the considerable avidity increase should be attributed to the multivalency effect, possibly based on statistical rebinding, which may result in stabilized ligand-lectin crosslinked complexes [38]. In sum, multivalent ligands, even with simple epitopes, such as the readily available lactose, are very promising as tools for biomedical research on Gal-4. The detailed mechanisms of multivalence and the avidity increase with multivalent ligands will be the subject of further studies.

## 4. Materials and Methods

### 4.1. Materials

Lactose (**1**) was from Lachema (Brno, Czech Republic). 2′-Fucosyllactose (**2**) was purchased from Biosynth (San Diego, CA, USA). Lacto-*N*-tetraose (**3**), lacto-*N*-neotetraose (**4**), blood-group antigen A type 6 (**5**), and blood-group antigen B type 6 (**6**) came from Elicityl (Crolles, France; in the catalog, they are rather uncommonly denoted as type 5). Protein-assay-dye-reagent concentrate and bovine plasma γ-globulin (IgG) for protein calibration were from BioRad (Watford, Hertfordshire, UK). *p*-Nitrophenyl β-d-galactopyranoside (*p*NP-Gal), 3,4-diethoxy-3-cyclobutene-1,2-dione (squaric acid diethyl ester), and Dowex 66 base-free were purchased from Sigma-Aldrich (Prague, Czech Republic). Buffers for ELISA assays were prepared from salts bought from Roth (Karlsruhe, Germany). If not stated otherwise, chemicals, solvents, general buffers, and cultivation media were purchased from VWR (Stříbrná Skalice, Czech Republic).

### 4.2. Synthesis of Carbohydrate Ligands of Gal-4

#### 4.2.1. Enzymatic Synthesis of Thomsen–Friedenreich Antigen (**9**; TF antigen; β-d-galactopyranosyl-(1→3)-2-acetamido-2-deoxy-d-galactopyranose)

The gene of wild-type β3-galactosidase BgaC from *Bacillus circulans* cloned in the vector pET-DuetTM-1 (*BamH*I/*Pst*I) was a kind gift from Prof. L. Elling, RWTH Aachen, Germany [39]. The enzyme was produced and purified essentially as described previously [40]. Briefly, *E. coli* BL21 Gold(DE3) cells (Takara Bio., Shanghai, China) were transformed with pET-Duet-1-bgaC plasmid. After inoculation in LB medium (60 mL in 0.5-L flasks) with 100 μg/mL ampicillin, transformed cells were shaken at 37 °C and 220 rpm overnight. The TB medium (600 mL in 3 L flasks; 24 g/L yeast extract, 12 g/L tryptone, 4 mL/L glycerol, 17 mM KH_2_PO_4_, 72 mM K_2_HPO_4_ pH 7.5, 100 μg/mL ampicillin) was inoculated with the preculture and incubated at 37 °C and 150 rpm until OD_600_ of 0.6–0.8. Enzyme expression was induced with 0.5 mM IPTG (isopropyl 1-thio-β-d-galactopyranoside, Merck, Darmstadt, Germany) and the cultures were grown at 25 °C and 150 rpm for 24 h. Cells were harvested by centrifugation (8,880× *g*, 20 min, 4 °C), frozen, and stored at −20 °C. For purification, cells were suspended in equilibration buffer (20 mM phosphate, 500 mM NaCl, 20 mM imidazole pH 7.4) with PMSF (phenylmethylsulfonyl fluoride, Merck, Darmstadt, Germany) (1 mM) and sonicated (6 cycles of 1 min of pulse and 2 min of pause). The lysate was centrifuged (20,230× *g*, 20 min, 4 °C) and BgaC was purified from the supernatant by affinity chromatography on the HisTrap™ column (Cytiva Life Sciences, Chicago, IL, USA); elution buffer: 20 mM phosphate/500 mM NaCl/500 mM imidazole pH 7.4). Purified BgaC (68.5 kDa) was pooled, dialyzed against 2 × 5 L of 50 mM sodium phosphate buffer with pH 6.5, and stored at 4 °C. The amount of 1g of cells afforded 134 mg of pure BgaC, as calculated by Bradford assay calibrated for IgG. Its activity was 1.2 U/mg, as determined by the end-point spectrophotometric assay with 2 mM *p*NP-Gal substrate in 100 mM acetate buffer pH 5 at 35 °C).

The glycosyl donor *p*NP-Gal (**7**; 45.2 mg, 0.15 mmol) and the acceptor *N*-acetylgalactosamine (**8**; 331.8 mg, 1.5 mmol) were dissolved in 100 mM acetate buffer pH 5.0 containing 20% *v*/*v* acetonitrile, and BgaC (0.25 U) was added (total reaction volume 5 mL) at 35 °C and 850 rpm. The reaction was monitored by TLC (isopropyl alcohol/H_2_O/NH_4_OH *aq*., 7/3/1, *v/v/v*; Rf = 0.26). When the donor was consumed (ca after 6 h), the reaction was stopped by enzyme denaturation (99 °C, 2 min), centrifuged at 12,100× *g*, and the supernatant was purified by gel-permeation chromatography (Biogel P2, 6.7 mL/h, water). After pooling and lyophilization, the title compound **9** was obtained as a white fluffy solid. The isolated yield was 31 mg (0.08 mmol, 51% isolated yield). The structural identity of **9** was confirmed by ^1^H NMR and ^13^C NMR (for methodology, see Heine et al. [41]), as shown in Supplementary Materials, Table S1, Figures S2 and S3). HPLC analysis is shown in Figure S5. MS (ESI^−^): *m*/*z* calculated 383.2 for C_14_H_25_NO_11_ was found to be 418.0 for [M + Cl]^−^ (Figure S4).

#### 4.2.2. Chemo-Enzymatic Synthesis of Lactosyl-Decorated Neo-Glycoprotein **12**

The *t*Boc-protected amino-functionalized lactose ligand **10** (lactosyl-*t*Boc) was synthesized essentially as described previously, at a 23% overall yield [31]. Its structural identity was confirmed by ^1^H NMR, and ^13^C NMR (for methodology, see Heine et al. [41]), as shown in Supplementary Materials, Table S2, Figures S6 and S7). HPLC analysis is shown in Figure S9. MS (ESI^+^): *m*/*z* calculated 543.2 for C_20_H_37_N_3_O_12_S, was found to be 544.2 for [M + H]^+^, 566.2 for [M + Na]^+^, 582.2 for [M + K]^+^ (Figure S8).

Lactosyl-*t*Boc **10** (10 mM) was deprotected in 1M HCl at 4 °C for 48 h to obtain the free amine. The reaction was neutralized with Dowex 66 base-free. Then, the ligand was coupled via its free amine to squaric acid diethyl ester (10 mM deprotected ligand, 40 mM squaric acid diethyl ester, 40 mM triethylamine, 35 mM Hepes pH 7.0/50% *v*/*v* ethanol) to afford **11**. After purification by HPLC (reversed-phase analytic MultoKrom 100-5 C18 column, 250 × 4.6 mm (CS Chromatographie, Langerwehe, DE) with 85/15 H_2_O/acetonitrile mobile phase, 1 mL/min), squarate monoamide ester **11** was isolated. MS (ESI^+^): *m*/*z* calculated 567.17 for C_21_H_33_N_3_O_13_S, was found to be 590 for [M + Na]^+^ (Figure S10). Next, **11** (2.5 eq.) was coupled to HSA (4 mg/mL, 50 mM tetraborate buffer pH 9.0) with shaking at 500 rpm and 20 °C for 72 h. Lactosyl-decorated neo-glycoprotein **12** was washed with water by ultracentrifugation and analyzed by MALDI-TOF (Supplementary Materials, Figure S11; for methodology, see Heine et al. [41]): 72,151 Da; 9.6 lactosyl residues. Its purity was confirmed by SDS-PAGE (8% gel).

### 4.3. Expression and Purification of Gal-4

The gene of human Gal-4 (GenBank: U82953.1; [42]) containing an N-terminal His_6_-tag cloned into the pET28a vector using the 5′-*Nde*I and 3′-*Xho*I restriction sites was obtained commercially (Generay, Shanghai, China). Gal-4 was expressed intracellularly in *Escherichia coli* Rosetta (DE3) pLysS cells (Merck, Darmstadt, Germany), first in an overnight preculture (60 mL, Luria-Bertani medium, 37 °C, 220 rpm), which was then transferred in TB (Terrific Broth, 600 mL in a 3-L flask) medium, both under pressure of kanamycin and chloramphenicol. When the culture reached an OD_600_ of 0.6, the expression of Gal-4 was induced by 0.5 mM IPTG. After 24 h of cultivation at 37 °C, the cells were harvested by centrifugation and frozen at −20 °C overnight (frozen cells could be stored for several months). On the next day, the cells were resuspended in 20 mL of binding buffer (20 mM Na_2_HPO_4_/500 mM NaCl/20 mM imidazole, pH 7.4) with 200 µL PMSF and disrupted by sonication (6 × 1 min), followed by centrifugation to remove the cell debris. The collected supernatant was diluted to the final 50 mL volume with binding buffer and loaded onto an equilibrated 5 mL HisTrap column (Cytiva Life Sciences, Chicago, IL, USA) connected to the Äkta Purifier protein-chromatography system (Cytiva Life Sciences, Chicago, IL, USA). The proteins bound to the column were eluted by the gradient (10 mL) of the elution buffer (20 mM Na_2_HPO_4_/500 mM NaCl/500 mM imidazole, pH 7.4) and the fractions containing Gal-4 (as determined by SDS-PAGE) were pooled and dialyzed against 7 L of EPBS (50 mM Na_2_HPO_4_/150 mM NaCl/2 mM EDTA, pH 7.5) overnight, and then against 7 L of PBS (150 mM NaCl/50 mM Na_2_HPO_4_, pH 7.5) for additional 4 h to remove traces of imidazole. The concentration of the prepared Gal-4 was determined using Bradford assay (calibrated for IgG), and its purity was determined by SDS-PAGE. The purified Gal-4 was stored at 4 °C for ca 3 weeks without a significant loss of activity. The identity of the His-tagged galectin constructs was confirmed using Western blot. The gel was transferred onto a nitrocellulose membrane after SDS-PAGE, and then blocked with 10% skimmed milk and labeled with anti-His_6_ antibody conjugated to HRP (His-probe, Santa Cruz Biotechnology, Dallas, TX, USA). The chemiluminescence signal was detected using G:box Chemi XRQ (Trigon Plus, Čestlice, Czech Republic) after substrate addition.

### 4.4. Expression and Purification of the N- and C-Terminal Subunits of Gal-4

The N-terminal subunit of Gal-4 (Gal-4NL) was identified as amino acid (aa) 1-150 in the original gene; the construct for expression contained additional 10 aa residues from the linker interconnecting both subunits at the C-terminus. The gene of Gal-4NL was prepared from the original plasmid containing the Gal-4 gene by PCR (T-Personal PCR cycler, Biometra, Göttingen, Germany) using Q5 Hot-Start High-Fidelity DNA polymerase (New England Biolabs, Ipswich, MA, USA) and the following primers: forward 5′-AGCTAGCTCATATGGCCTATGTCCCCGCACCGGGCT-3′ and reverse 5′-TATACGATCTCGAGTCAGGGTCCCTGGGGCCGGAG-3′. The PCR product and the original plasmid were subjected to cleavage by the *Nde*I and *Xho*I restriction endonucleases (New England Biolabs, Ipswich, MA, USA), purified by the GeneJET PCR Purification Kit (Thermo Scientific, Vilnius, Lithuania) and ligated using T4 DNA Ligase (New England Biolabs, Ipswich, MA, USA) at 16 °C, overnight. The ligation mixture was transformed into Gold(DE3) cells, the plasmids were isolated from the obtained colonies using the High Pure Plasmid Isolation Mini Kit (Roche, Basel, Switzerland), and the sequence of the construct was verified by commercial Sanger sequencing (SeqMe, Dobříš, Czech Republic).

The C-terminal-binding domain of Gal-4 (Gal-4CL) was identified as aa 179-323 in the original gene; the construct for expression contained an additional 10 aa residues from the linker interconnecting the two domains at the N-terminus. The Gal-4CL construct for expression was prepared analogously to the Gal-4NL described above, with the following PCR primers: forward 5′-AGCCAGTGCATATGCCCGGACATTGCCATCAACAGCT-3′ and reverse 5′-AGCACGATCTCGAGTCAGATCTGGACATAGGACAAGGTG-3′. The Gal-4NL and Gal-4CL subunits were expressed in *E. coli* Rosetta (DE3) pLysS and purified by the same procedure as described for the total Gal-4 in Section 4.3.

### 4.5. ELISA Assays with Gal-4 and Its Subunits

The potential of compounds 1–6, 9, and 12 to inhibit the binding of Gal-4 protein and its subunits to the immobilized standard competitor ASF was evaluated using competitive enzyme-linked immunosorbent assay (ELISA). Each incubation step was followed by washing the wells with 3 × 250 µL phosphate-buffered saline (PBS) containing 0.05% Tween^®^ 20. In total, 0.1 µM ASF in PBS (50 µL/well) was coated onto microtiter plates (Nunc Immuno Sorb, ThermoFisher Scientific, Waltham, MA, USA) overnight. After blocking the wells with 2 mg/mL bovine serum albumin (BSA) in PBS buffer (250 µL/well, 1 h), serial dilutions of the inhibitors in PBS buffer containing 2 mM EDTA (EPBS) were incubated with Gal-4 (2.5 µM final dimer concentration) or its subunits, Gal-4CL and Gal-4NL (25 µM final monomer concentration) for 2 h (50 µL/well). The residual bound galectin was labeled with an anti-His-antibody conjugated with horseradish peroxidase (HRP) (Santa Cruz Biotechnology, Heidelberg, Germany; 1:999 dilution in PBS). The colorimetric reaction with TMB One substrate solution (Kem-En-Tec, Taastrup, Denmark; 50 µL/well) was stopped with 3 M hydrochloric acid (HCl) (50 µL/well) and quantified spectrophotometrically at 450 nm (Sunrise; Tecan Group Ltd., Männedorf, Switzerland). The values of half-maximal inhibitory concentrations (IC_50_) were calculated by non-linear regression (dose–response inhibition-variable slope) of sigmoidal curves from a minimum of three independent experiments using GraphPad Prism 8.4.3 (GraphPad Software, San Diego, CA, USA).

The apparent dissociation constant (*K*_D_) of binding of the whole Gal-4 or its subunits to ASF was determined using direct enzyme-linked immunosorbent assay (ELISA), with washing steps as specified above. The microtiter plate was coated with immobilized ASF (0.1 µM) overnight and blocked with BSA (2 mg/mL, 250 µL/well) for 1 h. Increasing concentrations of Gal-4 or its subunits were added (50 µL/well) and incubated for 2 h. Residual galectins bound to the wells were labeled with anti-His-antibody conjugated with HRP, and evaluated as above.

### 4.6. Biolayer Interferometry (BLI)

The affinity and kinetics of neo-glycoprotein **12** with the whole Gal-4, Gal-4NL, and Gal-4CL were assessed by BLI under constant conditions (25 ± 0.1 °C, 800 rpm) using interferometry device Octet^®^ Red96e (FortéBio, Fremont, CA, USA). Galectins were diluted to a concentration of 2 µg/mL in PBS buffer containing 0.05% Tween-20 and immobilized on the Ni-NTA biosensor (Octet^®^ NTA Biosensors, Sartorius, Goettingen, Germany) via nickel chelation of N-terminal His-tags of the proteins. After the immobilization step (100 s), the interactions of the serially diluted (185–15 µM) neo-glycoprotein **12** with the immobilized proteins were monitored for 900 s during the association-and-dissociation step. No impairment of lectin activity due to immobilization and no non-specific interaction were observed. The acquired BLI data were investigated by Octet Analysis software (FortéBio, Fremont, CA, USA). The drift of the sensor itself, as obtained from the data with the reference sensor, was subtracted. The steady-state analysis was performed by plotting the shift of the interference pattern versus ligand concentration using Equation (1):

(1) $$R_{eq}=R_{max}\frac{c}{K_{D}+c}$$

where *R*_eq_ refers to the shift of the interference pattern at equilibrium in each sensogram curve, *R*_max_ refers to the maximal response at equilibrium, and *c* denotes the ligand concentration.

### 4.7. Dynamic Light Scattering (DLS)

The hydrodynamic diameter of the Gal-4 and its subunits (25 μM) was measured by dynamic light scattering (DLS) using Zetasizer Nano S90 (Malvern Instruments Ltd., Malvern, UK) at 25 °C. The light scattered at *θ* = 173° from the incident light was fitted to an autocorrelation function using the method of cumulants (Malvern Instruments Ltd., Malvern, UK). The hydrodynamic diameters were determined from five independent repetitions (10–50 runs) in Malvern disposable plastic cuvettes. The particle-size distribution was determined by analyzing the multimodal peak by intensity and volume.

## 5. Conclusions

This work presents a structure–affinity relationship study describing the affinities of the underappreciated Gal-4 protein with a series of carbohydrate ligands. It is the first study to numerically quantify the affinities of the entire Gal-4 protein and its two subunits with a variety of carbohydrate ligands. A robust and reliable ELISA design for assessing the affinities of this protein is presented. In addition, this work highlights the advantageous aspect of multivalency, which increased the avidity of a synthetic neo-glycoprotein for Gal-4 by more than three orders of magnitude. This result was validated by an alternative method of biolayer interferometry. In addition, the aggregation behavior of the whole Gal-4 and its subunits in solution was discussed, and its possible impact on the data obtained was outlined. These results represent a solid starting point for further biomedical and biological studies on the therapeutic or diagnostic inhibition/scavenging of Gal-4, such as in gastrointestinal cancer.

## Figures and Tables

**Table 1:** Yields of Gal-4 forms after purification by metal-ion-affinity chromatography.

Protein	Cells/L Medium [g/L]	Protein/Cells [mg/g]
Gal-4	1.8	4.2
Gal-4NL	2.2	2.1
Gal-4CL	3.1	2.4

**Table 2:** Inhibitory potency (IC_50_) of carbohydrate ligands to Gal-4.

Ligand	IC_50_ [mM]	*rp* ^a^
lactose (**1**) ^b^	2.5 ± 0.2	1.0
2′-fucosyllactose (**2**)	1.5 ± 0.2	1.7
lacto-*N*-tetraose (**3**)	0.43 ± 0.07	5.9
lacto-*N*-neotetraose (**4**)	0.6 ± 0.1	4.1
blood-group antigen A (**5**)	0.05 ± 0.02	48
blood-group antigen B (**6**)	0.08 ± 0.03	31
TF antigen motif (**9**)	1.18 ± 0.05	2.1
neo-glycoprotein **12**	0.00019 ± 0.00002	13,000 (1400) ^c^

^a^ *rp*, relative potency (*rp* = IC_50_ (lactose)/IC_50_ (ligand); ^b^ the IC_50_ value for lactosyl-*t*Boc **10** was 2.5 mM, and for the deprotected lactosyl amine, IC_50_ = 2.8 mM; ^c^ the value in the brackets expresses relative potency per lactosyl unit, i.e., *rp*/*lactosyl* = IC_50_ (lactose)/IC_50_ (ligand)/no. of lactosyls on the neo-glycoprotein, as determined by MALDI-TOF (9.6).

**Table 3:** Inhibitory potency (IC_50_) of carbohydrate ligands against Gal-4 subunits.

Ligand	IC_50_ [mM]		*rp* ^a^	
	Gal-4NL	Gal-4CL	Gal-4NL	Gal-4CL
lactose (**1**)	2.9 ± 0.4	8.6 ± 1.1	1.0	1.0
2′-fucosyllactose (**2**)	0.60 ± 0.06	1.1 ± 0.1	4.8	7.8
lacto-*N*-tetraose (**3**)	0.21 ± 0.03	0.47 ± 0.9	14	18.3
lacto-*N*-neotetraose (**4**)	0.4 ± 0.1	0.8 ± 0.3	7.3	10
Blood-group antigen A (**5**)	0.022 ± 0.001	0.027 ± 0.005	130	320
Blood-group antigen B (**6**)	0.26 ± 0.01	0.30 ± 0.03	11	28
TF antigen motif (**9**)	3.5 ± 0.2 ^b^	5.8 ± 0.5 ^b^	0.8	1.5
neo-glycoprotein **12**	0.0009 ± 0.0001	0.0017 ± 0.0004	3200 (340) ^c^	5100 (530) ^c^

^a^ *rp*, relative potency (*rp* = IC_50_ (lactose)/IC_50_ (ligand); ^b^ the value is an estimate from two independent experiments. ^c^ The value in brackets expresses relative potency per lactosyl unit, i.e., *rp*/*lactosyl* = IC_50_ (lactose)/IC_50_ (ligand)/no. of lactosyls on the neo-glycoprotein, as determined by MALDI-TOF (9.6).

**Table 4:** Hydrodynamic sizes of aggregates of Gal-4 and its subunits in solution measured by DLS.

Protein	Intensity-Weighed Size Distribution				Volume-Weighted Size Distribution		*PDI ^a^*
	Size [nm]	Area [%]	Size [nm]	Area [%]	Size [nm]	Area [%]	
Gal-4	11 ± 3	58	1200 ± 600	39	9 ± 2	100	0.2
Gal-4NL	1.3 ± 0.3	13	16 ± 7	50	3 ± 1	100	0.4
Gal-4CL	8 ± 3	63	400 ± 100	33	7 ± 2	100	0.2

*^a^ PDI*, polydispersity index expresses heterogeneity of the sample.

## Data Availability

The data are available from the corresponding author upon reasonable request.

## Supplementary Materials

The following supporting information can be downloaded at: https://www.mdpi.com/article/10.3390/molecules28104039/s1, Figure S1: Structures of β-d-Gal-(1→3)-d-GalNAc (**9**); (*t*-butoxycarbonylamino)ethylthioureidyl 2-acetamido-2-deoxy-β-d-galactopyranosyl-(1→3)-2-acetamido-2-deoxy-β-D-glucopyranoside (**10**); squarate monoamide ester **11**.; Figure S2: ^1^H spectrum of compound **9**; Figure S3: ^13^C spectrum of compound **9**; Figure S4: ESI-MS^+^ spectrum of compound **9**; Figure S5: HPLC chromatogram of compound **9**; Figure S6: ^1^H NMR spectrum of compound **10**; Figure S7: ^13^C NMR spectrum of compound **10**; Figure S8: ESI-MS spectra of compound **10**; Figure S9: HPLC chromatogram of compound **10**; Figure S10: ESI-MS^+^ spectrum of squarate monoamide ester **11**; Figure S11: MALDI-TOF spectrum of neo-glycoprotein **12**; Figure S12: SDS-PAGE analysis of isolated enzyme BgaC; Table S1: ^1^H and ^13^C NMR data of β-d-Gal-(1→3)-d-GalNAc **9**; Table S2: ^1^H and ^13^C NMR data of lactosyl-*t*Boc **10**.

## Sample Availability

Samples of the compounds and proteins used in this study are available from the authors.
