# Supplementary material for: Oligosaccharide Ligands of Galectin-4 and Its Subunits: Multivalency Scores Highly
Source: Molecules. 2023 May 11;28(10):4039. doi: 10.3390/molecules28104039 (PMC10223884; doi:10.3390/molecules28104039)
Supplement: Supplementary file 1 [file molecules-28-04039-s001.zip › molecules-2301102-supplementary.pdf]

Supplementary Materials

Oligosaccharide Ligands of Galectin-4 and Its Subunits: Multivalency Scores Highly

Kristýna Slámová [1], Jakub Červený [1,2], Zuzana Mészáros [1,3], Tereza Friede [1,4], David Vrbata [1], Vladimír Křen [1] and Pavla Bojarová [1,*]

[1] Laboratory of Biotransformation, Institute of Microbiology of the Czech Academy of Sciences, Vídeňská 1083, 142 00 Prague 4, Czech Republic; slamova@biomed.cas.cz (K.S.); cervenjaku@seznam.cz (J.Č.); kren@biomed.cas.cz (V.K.); david.vrbata@biomed.cas.cz (D.V.); zuzana.meszaros@biomed.cas.cz (Z.M.); tereza.friedova@gmail.com (T.F.)

[2] Department of Analytical Chemistry, Faculty of Science, Charles University, Hlavova 8, 128 43 Prague 2, Czech Republic

[3] Department of Biochemistry, University of Chemistry and Technology Prague, Technická 6, 160 00 Prague 6, Czech Republic

[4] Department of Biochemistry, Faculty of Science, Charles University, Hlavova 8, 128 43 Prague 2, Czech Republic

* Correspondence: bojarova@biomed.cas.cz; Tel.: +420-296-442-360

Analytical methods

NMR analysis

The NMR spectra of compounds **9** and **10** were recorded on Bruker Avance III 600 MHz (600.23 MHz for ^{1}H, 150.93 MHz for ^{13}C, D_2O, 30 °C) or 700 MHz (700.13 MHz for ^{1}H, 176.05 MHz for ^{13}C, D_2O, 30 °C). The performed experiments comprised ^{1}H NMR, ^{13}C NMR, gCOSY, ^{1}H-^{13}C gHSQC, ^{1}H-^{13}C gHMBC, HSQC-TOCSY, 1D-TOCSY. The signals were referenced as follows: protons to the residual signal of D_2O (δ_H 4.732 ppm), carbons to the signal of acetone (δ_C 30.50 ppm).

Mass spectrometry analysis

Electrospray-ionization mass spectrometry (ESI-MS) of compounds **9**, **10**, and **11** was performed using Shimadzu LCMS-2020 mass detector (Shimadzu, Japan). The samples were dissolved in acetonitrile (AcN/H_2O 4:1) and introduced into the mobile phase flow (acetonitrile) using a 2 µL loop. Spray voltage, capillary voltage, tube lens voltage, and capillary temperature were 4.0 kV, −16 V, −120 V, and 275 °C, respectively. Neo-glycoprotein **12** was analyzed by matrix-assisted laser desorption/ionization time-of-flight mass spectrometry. The sample was prepared by the dried droplet method. Each sample was diluted in 50% acetonitrile/ 0.1% TFA (10 pmol/µL). A saturated solution of sinapinic acid in 50% acetonitrile/ 0.1% TFA was added in equal volume to the sample, mixed well, the mixture spotted onto the MALDI target and dried at ambient temperature. The MALDI TOF spectra were measured on UltrafleXtreme MALDI TOF/TOF mass spectrometer (Bruker Daltonics, Germany) with 1 kHz smartbeam II laser. The measurements were done in a positive linear mode, with the mass range 20~200 kDa. The accelerating voltage was set at 25 kV. Typically, spectra were obtained by accumulating 10 000 shots.

HPLC analysis

Compounds **9** and **10** were analyzed on a TSKgel Amide-80 column (250 × 4.6 mm, 5 µm, HILIC) preceded by TSKgel Amide-80 Guardgel (3.2 × 15 mm, Tosoh Corp., Japan) in acetonitrile/water (4/1, *v/v*), with gradient elution as follows (A= acetonitrile, B= water): 22% B for 0-7 min, 22-31% B for 7-16 min, 31-22% B for 16-17 min, and 22% B for 17-22 min for column equilibration. The flow rate was 1 mL/min, temperature 25 °C, injection volume 1 µL, the detection was performed at 200 nm. The Shimadzu Prominence LC analytical system comprised of Shimadzu LC-20AD binary HPLC pump, Shimadzu SIL-20ACHT cooling autosampler, Shimadzu CBM-20A system controller, Shimadzu CTO-10AS column oven, and Shimadzu SPD-20MA diode array detector (Shimadzu, Japan).

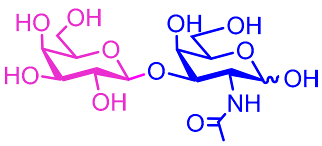

TF antigen motif (**9**)

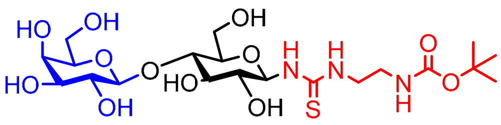

Lactosyl-*t*Boc (**10**)

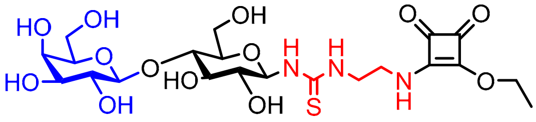

Lactosyl-squarate (**11**)

Figure S1. Structures of β-D-Gal-(1→3)-D-GalNAc (**9**); (*t*-butoxycarbonylamino)ethylthioureidyl 2-acetamido-2-deoxy-β-D-galactopyranosyl-(1→3)-2-acetamido-2-deoxy-β-D-glucopyranoside (**10**); squarate monoamide ester **11**. For details see the main text.

Table S1. ^{1}H a ^{13}C NMR data of β-D-Gal-(1→3)-D-GalNAc (**9**; 600.23 MHz for ^{1}H, 150.93 MHz for ^{13}C, D_2O, 30 °C).

α–anomer

	Atom #	δ_C	m.	δ_H	n_H	m.	J_{C-H} [Hz]	diagnosis of HMBC
**GalNAc**	**1**	91.49	d	5.279	1	d	3.8	
	**2**	49.27	d	4.357	1	dd	11.1, 3.8	
	**3**	77.34	d	4.088	1	dd	11.1, 3.1	**1′**
	**4**	69.00	d	4.302	1	d	3.1	
	**5**	70.47	d	4.193	1	m		1
	**6**	61.45	t	3.80^{H}	2	m		
	**2-CO**	174.93	s	-	0	-		
	**Ac**	22.33	q	2.088	3	s		
**Gal**	**1′**	104.90	d	4.554	1	d	7.8	**3**
	**2′**	70.97	d	3.587	1	dd	10.0, 7.8	
	**3′**	72.87	d	3.683	1	dd	10.0, 3.4	
	**4′**	68.88	d	3.974	1	d	3.4	
	**5′**	75.24	d	3.72^{H}	1	m		1′
	**6′**	61.24	t	3.82^{H}	2	m		

β–anomer

	Atom #	δ_C	m.	δ_H	n_H	m.	J_{C-H} [Hz]	diagnosis of HMBC
**GalNAc**	**1**	95.46	d	4.757	1	d	8.5	
	**2**	52.80	d	4.039	1	dd	10.9, 8.5	
	**3**	80.30	d	3.924	1	dd	10.9, 3.2	**1′**
	**4**	68.35	d	4.235	1	d	3.2	
	**5**	75.11	d	3.767	1	m		1
	**6**	61.24	t	3.82^{H}	2	m		
	**2-CO**	175.23	s	-	0	-		

	**Ac**	22.56	q	2.085	3	s		
**Gal**	**1′**	105.07	d	4.498	1	d	7.8	**3**
	**2′**	70.91	d	3.587	1	dd	10.0, 7.8	
	**3′**	72.82	d	3.673	1	dd	10.0, 3.4	
	**4′**	68.88	d	3.968	1	d	3.4	
	**5′**	75.26	d	3.72^{H}	1	m		1′
	**6′**	61.24	t	3.82^{H}	2	m		

^{H} … HSQC data

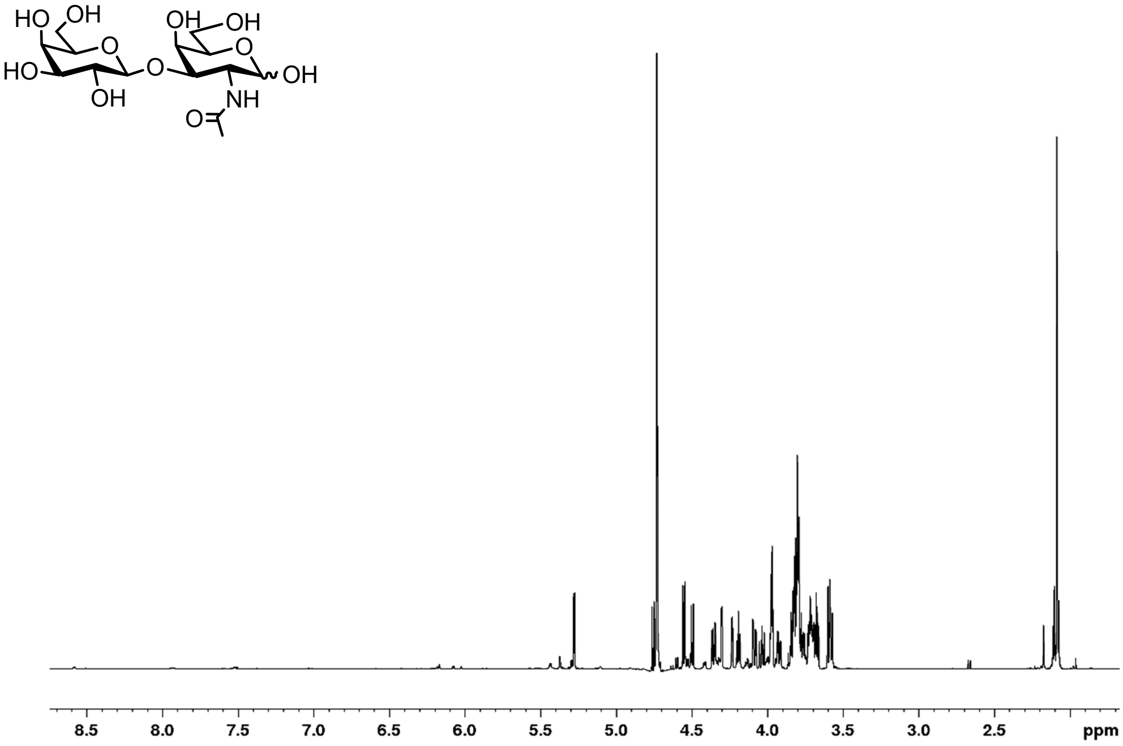

Figure S2. ^{1}H spectrum of compound **9** (600.23 MHz for ^{1}H, 150.93 MHz for ^{13}C, D_2O, 30 °C).

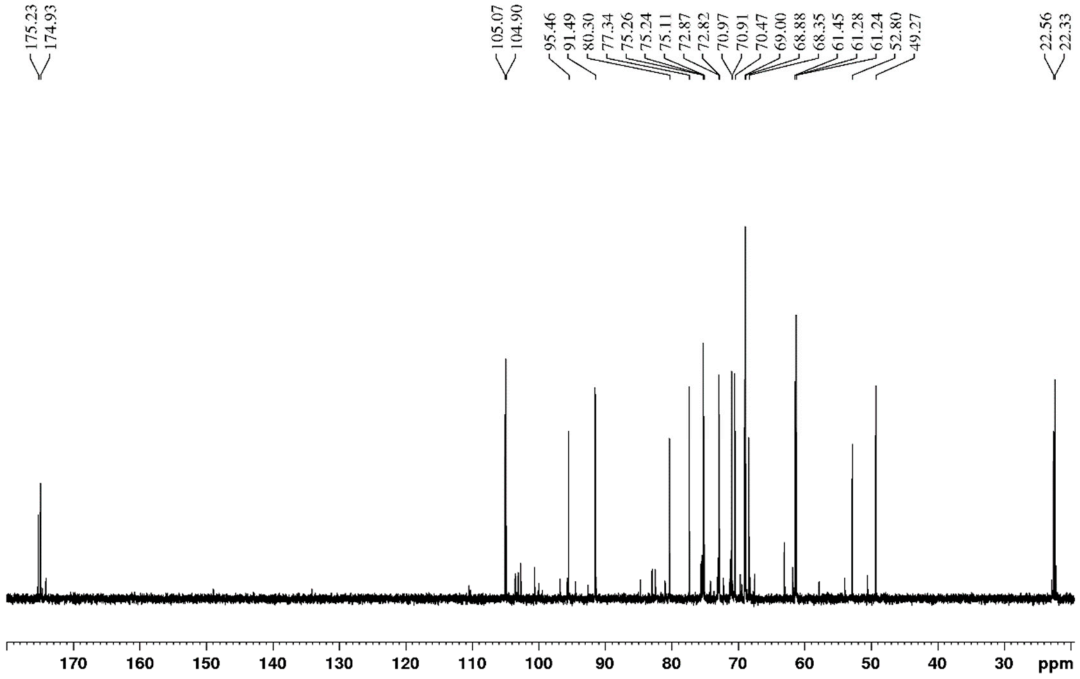

Figure S3. ^{13}C spectrum of compound **9** (600.23 MHz for ^{1}H, 150.93 MHz for ^{13}C, D_2O, 30 °C).

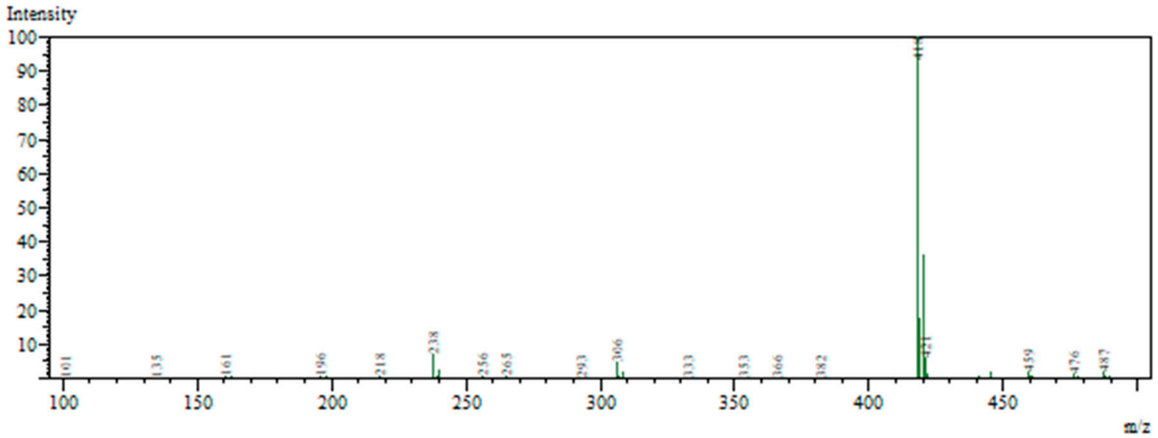

Figure S4. ESI-MS$^+$ spectrum of compound **9** (exact mass: 383.2; detected mass: $[M + Cl]^+$, *m/z* 418; $[M + Na]^{+}$

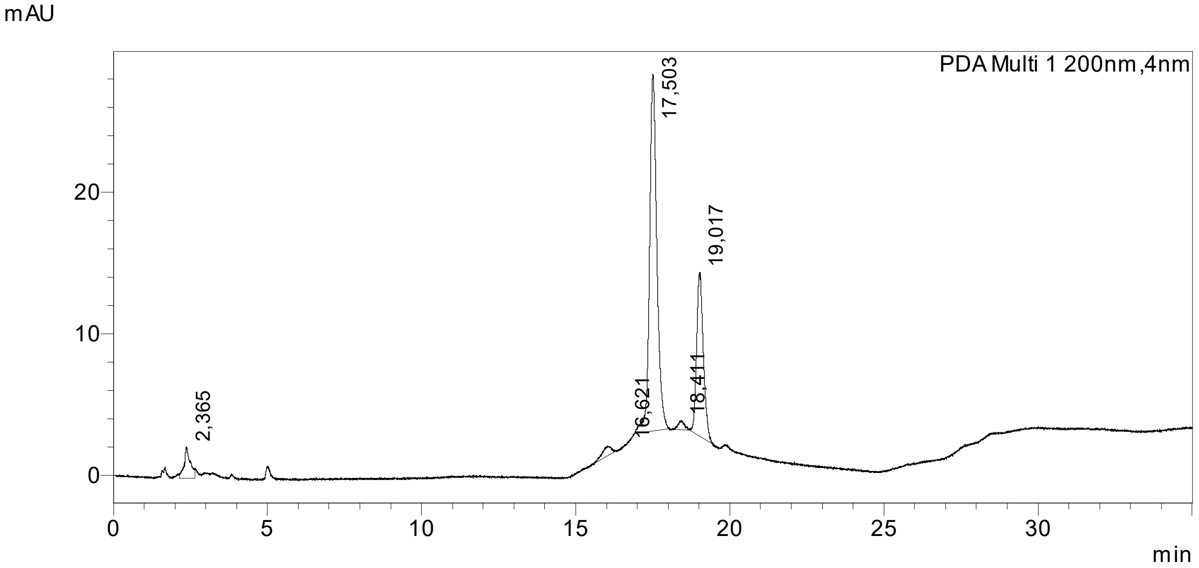

Figure S5. HPLC chromatogram of compound **9** (retention time 17.503 min for β-anomer; retention time 19.017 min for α-anomer). Purity 93%.

Table S2. ^{1}H a ^{13}C NMR data of lactosyl-*t*Boc (**10**; 700.13 MHz for ^{1}H, 176.05 MHz for ^{13}C, D_2O, 30 °C).

	**Atom**	**δ_C**	**m.**	**δ_H**	**n_H**	**m.**	***J* [Hz]**	**diagnostic HMBC**
**Boc**	**CO**	158.52	S	-	0	-		2′
	**C**	81.36	S	-	0	-		$(CH_3)_3$
	**$(CH_3)_3$**	27.95	Q	1.456	9	s		$(CH_3)_3$
**spacer**	**1′**	44.53	T	3.71^{H}	2	m		
	**2′**	39.57	T	3.318	2	br t		
	**CS**	n.d.	S	-	0	-		
**Glc^{A}**	**1**	83.38^{x}	D	5.34^{x}	1	br s		
	**2**	71.87	D	3.532	1	br m		
	**3**	75.29^{x}	D	3.72^{H}	1	m		
	**4**	78.12	D	3.71^{H}	1	m		1^{B}
	**5**	76.23	D	3.71^{H}	1	m		
	**6**	60.20	T	3.97^{H}	1	m		
				3.84^{H}	1	m		
**Gal^{B}**	**1**	103.13	D	4.485	1	d	7.9	
	**2**	70.25	D	3.625	1	dd	9.9, 7.9	
	**3**	82.27	D	3.754	1	dd	9.9, 3.3	1^{C}
	**4**	68.59	D	4.177	1	d	3.3	
	**5**	75.14	D	3.74^{H}	1	m		
	**6**	61.19^{a}	T	3.80^{H}	2	m		

^{H} … HSQC readout; ^{a,b} … might be interchanged; ^{x} … tentative assignment; n.d. … not detected

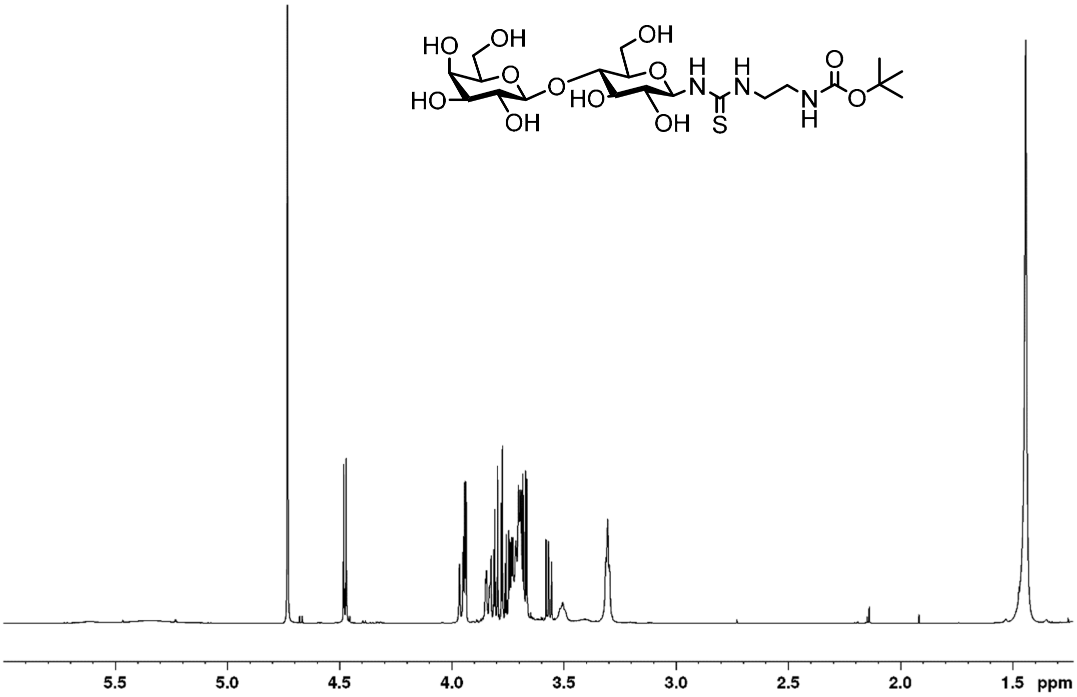

Figure. S6. ^{1}H NMR spectrum of compound **10** (700.13 MHz, D_2O, 30 °C).

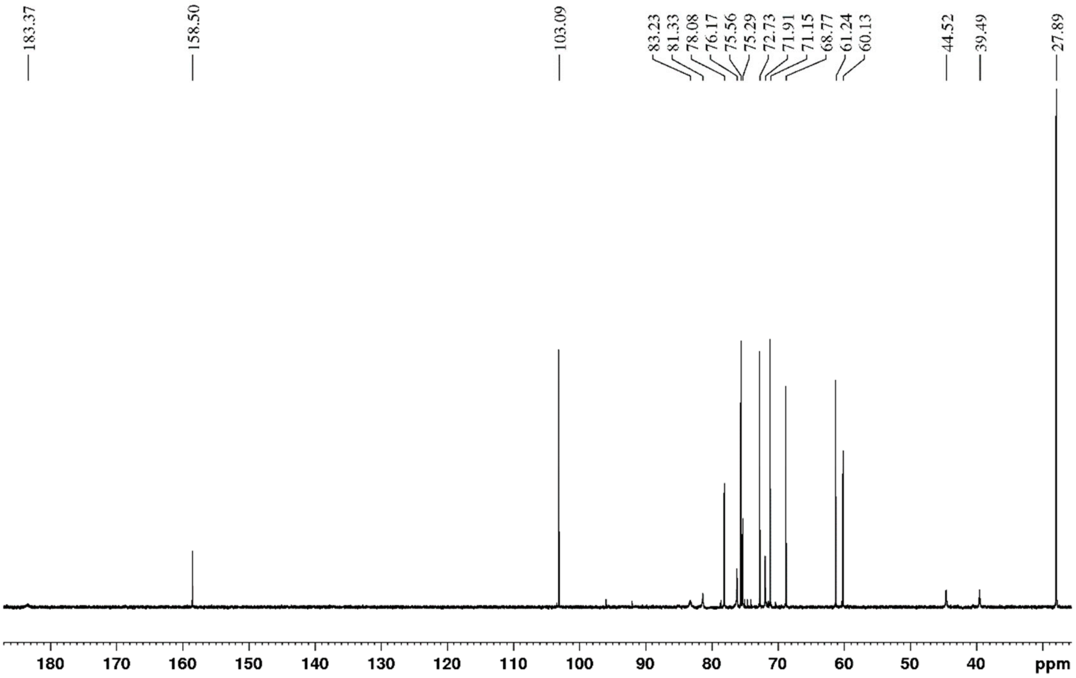

Figure S7. ^{13}C NMR spectrum of compound **10** (176.05 MHz, D_2O, 30 °C).

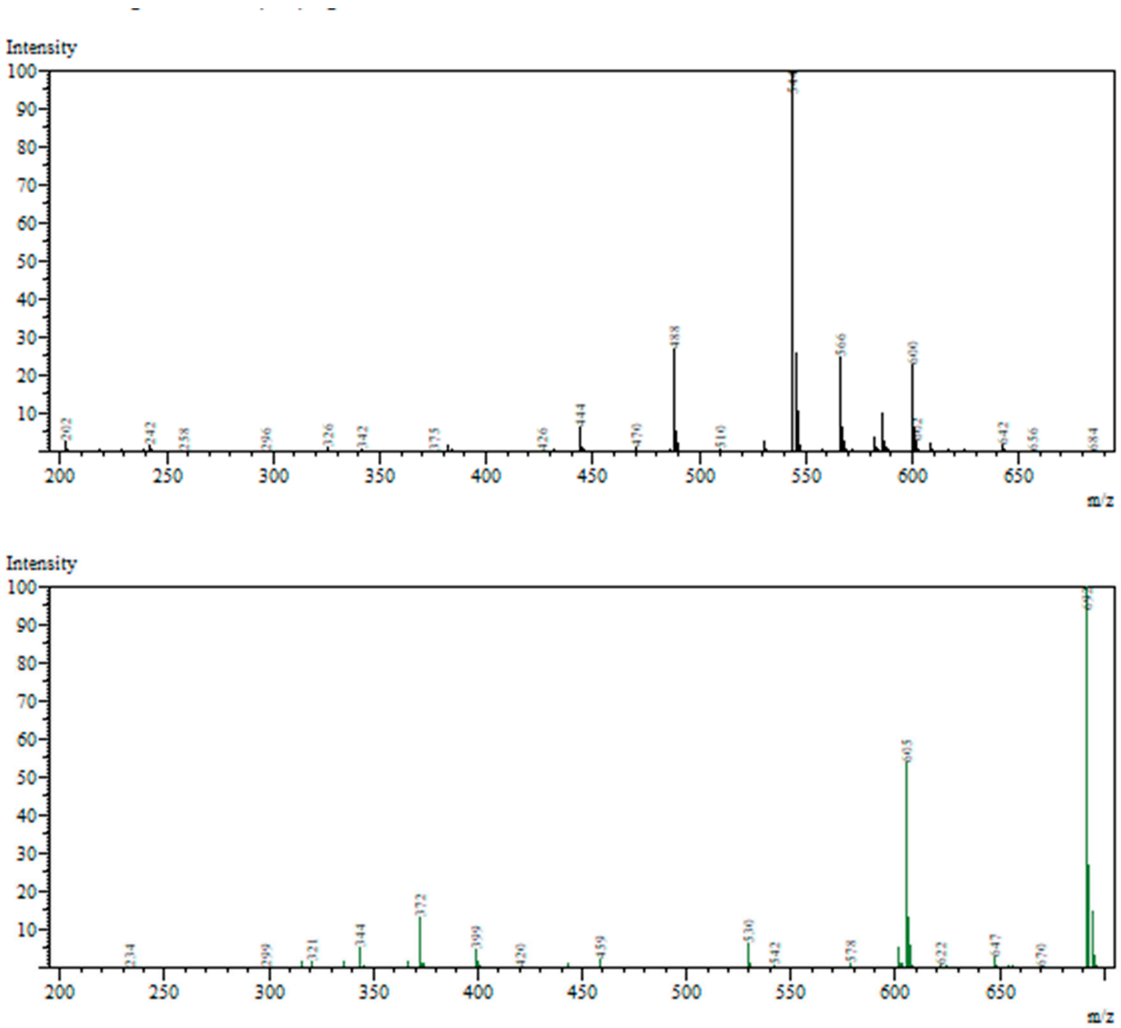

Figure S8. ESI-MS spectra of compound **10** (exact mass: 543.21; detected mass: $[M + H]^+$, *m/z* 544; $[M + Na]^+$, *m/z* 566; $[M + CH_3COO]^-$, *m/z* 605.

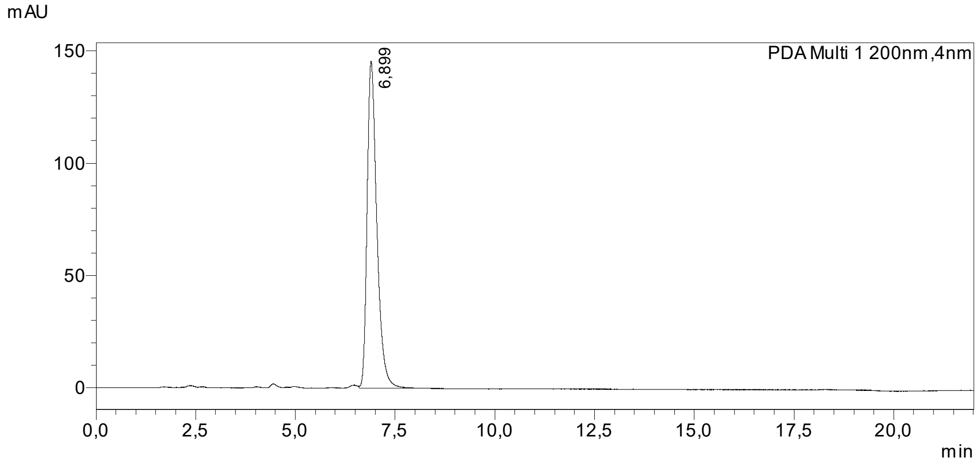

Figure S9. HPLC chromatogram of compound **10** (retention time 6.899 min). Purity 99%.

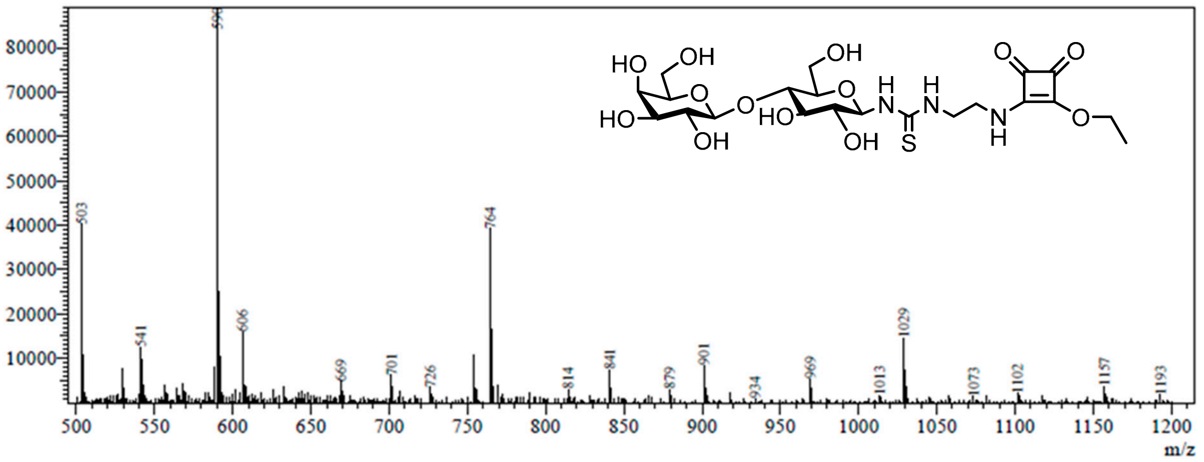

Figure S10. ESI-MS+ spectrum of squarate monoamide ester **11** (exact mass: 567.17; detected mass: $[M + Na]^+$, *m/z* 590).

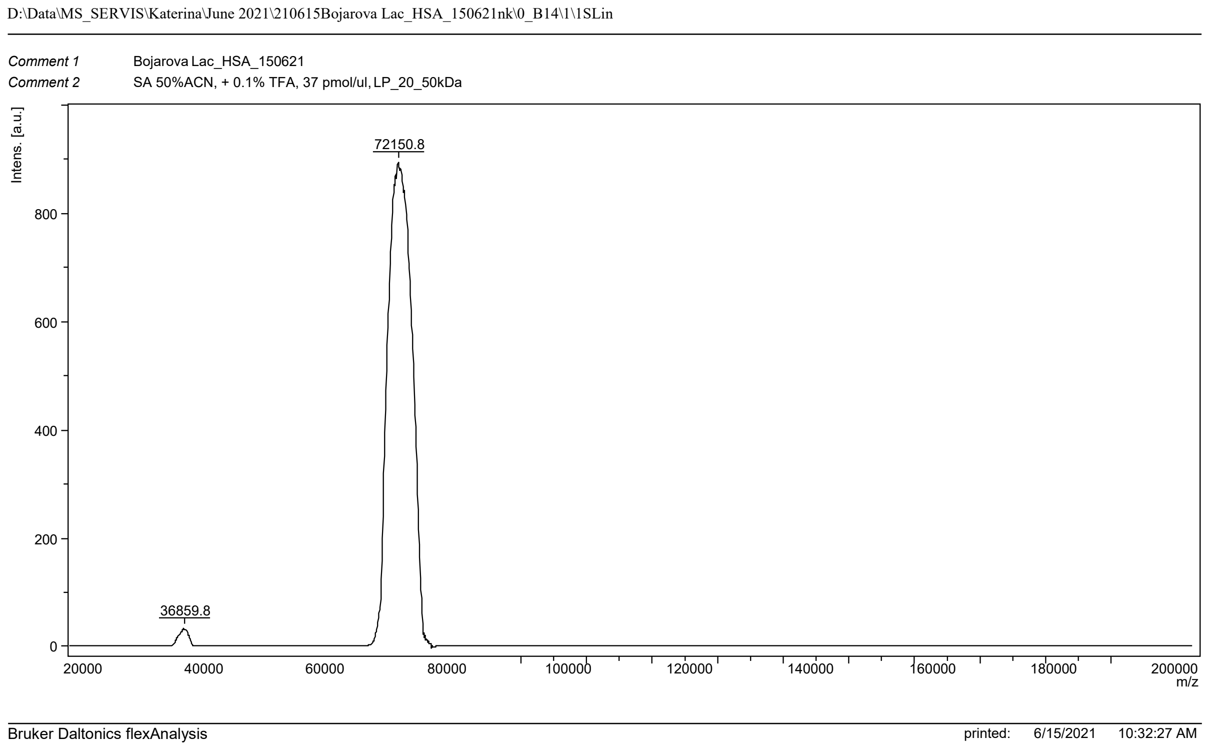

Figure S11. MALDI-TOF spectrum of neo-glycoprotein **12**.

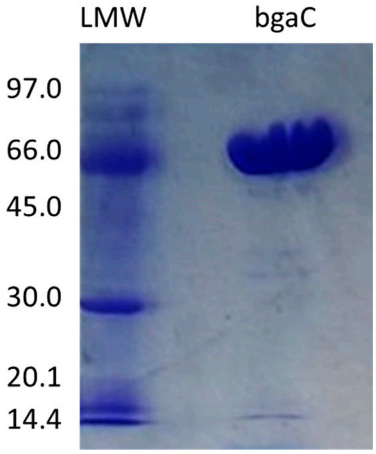

Figure S12. SDS-PAGE analysis of isolated enzyme BgaC (67 kDa). LMW – Amersham Low Molecular Weight Calibration Kit for SDS Electrophoresis (GE Healthcare, Chicago, USA): 97 kDa – phosphorylase b from rabbit muscle; 66 kDa – bovine serum albumin; 45 kDa – chicken egg-white ovalbumin; 30 kDa – carbonic anhydrase from bovine erythrocyte; 20.1 kDa – trypsin inhibitor from soybean; 14.4 kDa – α-lactalbumin from bovine milk.
